# A survey of Japanese quail (*Coturnix coturnix japonica*) farming in selected areas of Bangladesh

**DOI:** 10.14202/vetworld.2016.940-947

**Published:** 2016-09-07

**Authors:** Abu Nasar, Aminoor Rahman, Nazmul Hoque, Anup Kumar Talukder, Ziban Chandra Das

**Affiliations:** Department of Gynecology, Obstetrics & Reproductive Health, Faculty of Veterinary Medicine & Animal Science, Bangabandhu Sheikh Mujibur Rahman Agricultural University, Gazipur 1706, Bangladesh

**Keywords:** Bangladesh, Japanese quail, problems, prospects, status

## Abstract

**Aim:**

To investigate the status, problems and prospects of Japanese quail (Coturnix coturnix japonica) farming in selected areas of Bangladesh.

**Materials and Methods:**

The study was conducted in 14 districts of Bangladesh, viz., Dhaka, Narayanganj, Munshiganj, Mymensingh, Netrakona, Faridpur, Jessore, Khulna, Satkhira, Kushtia, Bogra, Naogaon, Comilla, and Sylhet during the period from July 2011 to June 2012. A total of 52 quail farmers were interviewed for data collection using a structured questionnaire. Focus group discussions were also carried out with unsuccessful farmers and those want to start quail farming. Workers of quail farms, quail feeds and medicine suppliers, quail eggs and meat sellers were also interviewed regarding the issue.

**Results:**

Out of 52 farms, 86.5% were operated by male, 67.3% farmers did not receive any training and 92.3% farmers had no earlier experience of quail farming although 58.0% farmers primary occupation was quail farming. Most of the farms (63.4%) were mixed in type having ≤5000 birds of two or three varieties. About 80.7% farms were operated separately round the year with no other poultry and 83.0% farmers wanted to expand their farming. The average pullet weight 145.0±0.12, 110.0±0.07, 120.0±0.22, and 128.0±0.17 g; age at the first lay 46.0±0.04, 42.0±0.31, 42.0±0.09, and 45.2±0.05 days; rearing period 15.0±0.01, 12.0±0.14, 15.0±0.32, and 15.2±0.18 months; culling period 15.5±0.14, 13.0±0.06, 15.0±0.03, and 15.4±0.26 months were for layer, parent stock, hatchery, and mixed farms, respectively. Most of the layer farms had an average egg production of ≤5000/day and net profit BDT 0.75/egg. However, an average number of birds, hatchability and net profit per day-old-chick were ≤5000, 76.8% and BDT 2.75, respectively, in the hatchery. Broiler quails were sold at 30 days with mean weight of 110.8 g and net profit BDT 9.02/bird. The major constraints of quail farming were higher feed price, outbreak of endemic diseases, lack of proper knowledge, farmers training, proper market access, difficulties of parent stock collection, inadequate biosecurity practices, and limited access to veterinary care. Thus, a proper training on quail farming, bio-security management, and government subsidy on feeds could make quail farming sustainable in Bangladesh.

**Conclusions:**

The study concludes that Japanese quail farming has enormous potentiality and could be an alternative to chicken farming particularly in providing gainful employment, supplementary income and as a valuable source of meat and egg, quail farming should be encouraged and promoted in Bangladesh.

## Introduction

Bangladesh is an agriculture-based developing country with approximately 140 million poultry [1]. The majority of these poultry are indigenous chickens and ducks [2]. The productive performance of this chickens is low and losses due to diseases and predators are high [3]. However, exotic pure breeds did not perform satisfactorily in scavenging system because of their higher nutritional demand and lower disease resistance [4]. Therefore, in addition to indigenous poultry, rural and semi-urban people need such a suitable species of bird which can be reared easily with little investment and provide more economic return within a very short time.

Quails were small game birds that are now used for commercial production of eggs and meat [5], and they attain rapid sexual maturity have shorter incubation period and can produce up to four generations per annum, therefore making them the most suitable and effective poultry [6]. There are two species of quails suitable for breeding, *viz*., the Japanese quail (*C. coturnix japonica*) and the American or common quail (*C. coturnix*). Japanese quails belong to Phasianidae family and are migratory birds which migrate between Asia and Europe [5,6]. Japanese quails are the smallest member of poultry with immense potentiality [7], and therefore, used in commercial production for meat and egg [8,9]. Egg production is important in Far East and Asian countries, whereas meat production is important in Europe [10].

The Japanese quails are blessed with many desirable characteristics, *viz*., faster growth, early sexual maturity, high rate of egg production (300 egg/annum), short generation interval (3-4 generations a year), small floor space (200-250 and 150-200 cm^2^, respectively in litter and cage system), less feed requirements (20-25 g/adult bird/day), short incubation period of hatching eggs, less feed cost, and less susceptibility to common chicken diseases [11-13]. Because of these encouraging economic traits, quail farming needs much lower capital investment as compared to chicken and duck with almost the same profit margin [7,13]. Japanese quail eggs have a high potential to be developed as a cheaper source of protein, especially in developing countries. In addition to being cheaper and delicious, quail eggs are rich in protein and good sources of folate, vitamin B_12_, pantothenic acid, iron, phosphorus, riboflavin and selenium [14]. Quail meat is tender, tasty, nutritious, and gaining popularity as a table delicacy among the consumers [15]. In regard to meat quality (pH, color, and texture), the quail meat is similar to broiler meat [16]. The nutritional value of quail eggs is 3-4 times greater than chicken eggs since it contains more moisture, minerals than broiler meat and has less fat and fewer calories, forming an ideal food for health conscious consumers [15,17]. Due to its small size and short generation interval, the Japanese quail is also popular as laboratory animal [18].

Although, compared to chicken it is quite a new species of poultry in Bangladesh, however, it has already received renewed impetus with the passage of time [19]. Its immense potentialities as a new dimension in poultry farming have already been recognized in this country. However, till now, the status and the major problems of quail farming in Bangladesh are not properly explored. Therefore, the study was undertaken to obtain thorough and detailed information on the status, problems, and prospect of Japanese quail farming in selected areas of Bangladesh.

## Materials and Methods

### Ethical approval

All the procedures of the study were performed under the approval of Bangabandhu Sheikh Mujibur Rahman Agricultural University’s Animal Experimentation Ethics Committee.

### Study area and farm management

**Figure 1:**
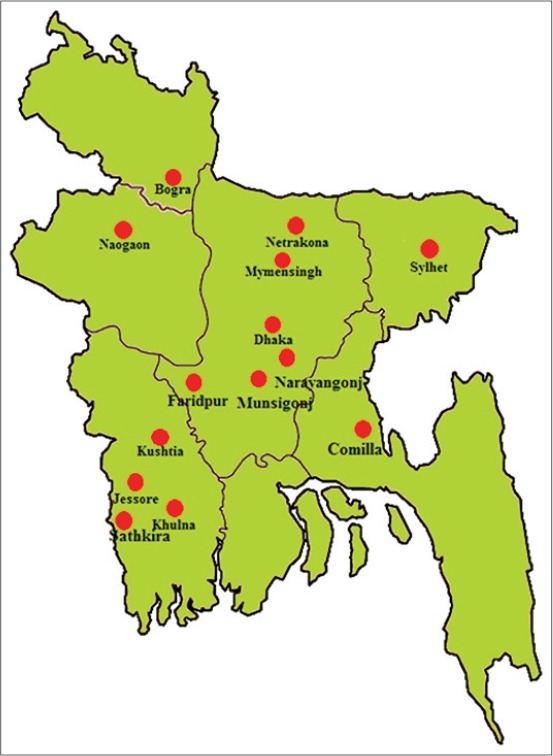
Distribution of Japanese quail farms in Bangladesh.

The study was conducted in 14 districts of Bangladesh, *viz*., Dhaka, Narayanganj, Munshiganj, Mymensingh, Netrakona, Faridpur, Jessore, Khulna, Satkhira, Kushtia, Bogra, Naogaon, Comilla, and Sylhet during the period from July 2011 to June 2012 (Figure-1). Bangladesh is vulnerable due to its position in the globe (Southeast Asian country). The climate is tropical; mild winter (October to March); hot, humid summer (March to June); humid, warm rainy monsoon (June to October). The country’s total land area is about 147,570 km^2^, and geographic position is latitude: 20°45’ to 26°40’N, longitude: 88°05’ to 92°40’E. The average annual rainfall varies from a maximum of 5690 mm in the northeast of the country to minimum of 1110 mm in the west. The day temperature ranges from 7 to 12°C in the cool months, and in the other months, it varies between 23 and 30°C. All the farm houses were built using brick, net and tin, and floor rearing system was practiced for birds of all ages (brooding, growing and laying).

### Data collection

The samples of this study were quail farmers (small, medium and large), including successful farmers, unsuccessful farmers those have already closed their farms, new farmers and interested peoples who want to start quail farming. A structured questionnaire schedule was prepared for interviewing the quail farmers in the selected areas of Bangladesh. This questionnaire was first piloted on a very small group of farmers, and then the questions were restructured in accordance with their responses. Focus group discussions were also carried out with unsuccessful farmers and those want to start quail farming. Workers of quail farms, quail feeds and medicine suppliers, quail eggs and meat sellers were also interviewed regarding the issue.

### Statistical analysis

The data generated from this experiment were entered in Microsoft Excel (2007) worksheet, organized and processed for further analysis. Analysis was performed with the help of Statistical Packages for Social Sciences, version 11.5 for windows (SPSS, Inc., Chicago, IL, USA). Descriptive statistics were computed and histogram is drawn to figure out the net profit (gross income-gross cost) for individual circumstance.

## Results and Discussion

**Figure 2:**
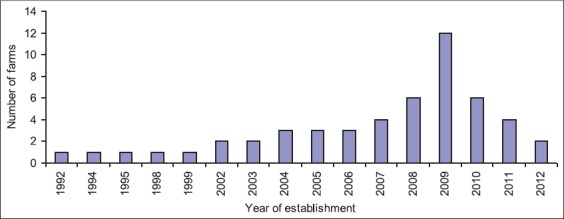
Establishment of quail farms in Bangladesh during the period from 1992 to 2012 [2,8,41].

To the best of our knowledge, there are no studies available on the present scenario, prospects and major constraints of Japanese quail farming in Bangladesh and this one might be the first ever study in this regard. The study was carried out in 52 quail farms and among these, 45 farms (86.5%) were operated by male and quail farming was the primary occupation for 58.0% (30/52) farm holders. Most of the farmers (69.2%; 36/52) neither read any book on quail farming nor played any significant role in spreading the industry by motivating the jobless youth and distressed women providing training and technical support. The study also revealed that 67.3% farm holders did not receive any training and 92.3% farmers had no earlier experience of quail farming. Quail farming in Bangladesh was started in 1992 and remained static for about one decade (1992-2003) since its inception, thereafter, gradually increased till 2009 (highest in 2009) and gradually declined thereafter (Figure-2). The reasons might be outbreak of epidemics, faulty management systems, higher price of feeds, the higher incidence of different infectious diseases, and lack of veterinary care.

The detail status of quail farming in Bangladesh has been shown in Table-1. Majority of the farmers (63.4%) had a practice of mixed type quail farming. However, layer, parent stock and broiler or meat type farming were only practiced by 21.1%, 3.8% and 9.6% farmers, respectively. Mixed type quail farming is practiced worldwide because Japanese quails are suited for commercial rearing for egg and meat production under intensive management [20]. This is because of their hardiness and ability to thrive in small cages [21]; the relative short generation interval and cheaper cost of production [22]. In our study, about 78.8% farmers did not rear quail with other poultry. Most of the farmers had ≤5000 birds with 2-3 varieties and reared quails separately. About 80.7% farms were operated round the year and 83.0% farmers wanted to expand their farming in future. The average pullet weight was 145.0±0.12, 110.0±0.07, 120.0±0.22 and 128.0±0.17 g for layer, parent stock, hatchery and mixed farms, respectively. The average age at the first lay was 46.0±0.04, 42.0±0.31, 42.0±0.09, and 45.2±0.05 days; rearing period was 15.0±0.01, 12.0±0.14, 15.0±0.32, and 15.2±0.18 months; culling period 15.5±0.14, 13.0±0.06, 15.0±0.03, and 15.4±0.26 months for layer, parent stock, hatchery and mixed farms, respectively.

**Table-1:** Status of quail farming (n=52) in Bangladesh.

Criteria	Groups	n (%)
Farm type	Layer	11 (21.1)
	Parent stock	2 (3.8)
	Broiler/meat type	5 (9.6)
	Hatchery	1 (1.9)
	Mixed type	33 (63.4)
Whether reared with other poultry	Yes	11 (21.1)
	No	41 (78.8)
Farm size (number of quails)	<1000	4 (7.7)
	1001-2500	15 (28.8)
	2501-5000	17 (32.7)
	5001-10,000	7 (13.4)
Number of breeds/varieties	One breed/variety	1 (1.9)
	Two breeds/varieties	17 (32.7)
	Three breeds/varieties	13 (25.0)
	Four breeds/varieties	7 (13.4)
Pattern of rearing	Together	22 (42.3)
	Separately	30 (57.7)
Farm operation	Year round	42 (80.7)
	Seasonal	10 (19.2)
Future plan	Want to expand farm	39 (83.0)
	Want to stop farming	8 (17.0)

Most of the layer farms (95.2%) had an average egg production of ≤5000/day (Table-2). In a recent study, Singh [23] reported that on an average Japanese quail weighs about 8-9 g from hatching egg weight ranges from 10 to 12 g, average body weight at 5-6 weeks is 180-200 g, and adult body weight is 200-250 g. However, there might be a significant variation in all the laying parameters among different local and imported stocks of Japanese quails [24]. The domestic quail shows rapid growth and attains sexual maturity at 5-6 weeks of age. Nowadays, meat type (broiler) quail strains are slaughtered at 5 weeks of age with a weight of 160-250 g [18,24]. Females enter into full lay at about 8-9 weeks of age. Layers are usually kept up to 8-10 m of age and produce about 300 eggs per year each with a weight of 7-11 g [18,25].

Detailed information on parent stock or broiler type quail farming has been shown in Table-3. Most of the farmers (47.8%; parent stock and 57.5%; broiler farms) have collected day-old-chick from their own farms. The parent stock farms had an average population of 7872 birds/farm with an average egg production of 5510/day (production rate 70.0%) and male-female ratio found in most cases was 1:3. 65.0% parent stock farmers used incubator made from other sources having hatchery capacity of >5000 eggs/incubator and the average hatchability was 76.8% which was lower than the hatchability reported 84.4-91.8% based on different incubation methods [12,26]. However, there were no significant differences in egg weight loss, hatchability, embryonic mortality, supply organ weights, spread of hatch or relative growth in relation to different treatment methods or incubation techniques [27]. Recent studies have shown that the mate choices of female quail are influenced by prior observations of males interacting with either females or other males as well as by direct interaction with males [25,28]. However, Gebreil [29] emphasized that for the optimum fertility male-female ratio must be between 1:1 and 1:3 for Japanese quails. In another study, Narinc *et al*. [30] observed the highest percentage of fertility in 1:1 (92.21%) and 1:2 (91.18%) male-female mating ratio. Nevertheless, it has also been reported in several studies that fertility was decreased below 80% for 1:4 and 1:5 mating ratios [30,31]. The mate choices of female Japanese quail can also be influenced by the behavior of conspecific females [32].

**Table-2:** Patterns of layer type quail farming (n=42) in study areas.

Farming categories	Pullet weight (g)	Age at first lay (days)	Rearing period (months)	Age of culling (months)
Layer	145.0±0.12	46.0±0.04	15.0±0.01	15.5±0.14
Parent stock	110.0±0.07	42.0±0.31	12.0±0.14	13.0±0.06
Hatchery	120.0±0.22	42.0±0.09	15.0±0.32	15.0±0.03
Mixed	128.0±0.17	45.2±0.05	15.2±0.18	15.4±0.26
Egg production/day	<500	501-1000	1001-5000	5001-10000
	2 (4.8%)	16 (38.0%)	22 (52.3%)	2 (4.8%)

Data were presented as mean±SE. SE: Standard error

**Table-3:** Status of parent stock and broiler farming in selected areas of Bangladesh.

Criteria	Groups	n (%)
Parent stock farming (n=23 farms)		
Collection of parent stock	Own farm	11 (47.8)
	Contact farm	7 (30.4)
	Others sources	5 (21.7)
Average number of parent stock/farm	7872 (500-50,000)	
Male female ratio	1:2	4 (17.4)
	1:3	17 (73.9)
	1:4	2 (8.6)
Average hatching egg production/day	5510 (350-15,500)	
Incubator source	Self made	8 (34.8)
	Other source	15 (65.2)
Hatcher capacity	2001-3000	1 (4.3)
	3001-4000	4 (17.4)
	4001-5000	1 (4.3)
	>5000	17 (73.9)
Average hatchability	76.8% (70-85)	
Broiler farming (n=33 farms)		
Type of farm	Broiler	5 (15.1)
	Meat type	23 (69.7)
	Others	5 (15.1)
Collection of day old chick	Own farm	19 (57.5)
	Contact farm	9 (27.2)
	Others	4 (12.1)
	Own+Contact farmer	1 (3.0)
Average marketing age (day)	30 (25-35)	
Average number of broiler/farm	5588 (700-31,000)	
Average weight (g) at slaughter	Undressed	110.8 (80-150)
	Dressed	76.93 (60-100)

In our current study, most of the broiler quail farms (69.7%) had an average size of 5588 birds/farm and the average marketing age of these birds was 30 days (Table-3). The average weight for broiler quail is 110.8 g at slaughter and 76.9 g after dressing. The female birds were heavier in weight than the male both at slaughter and after dressing. The weight of the broiler quails in this study was somewhat lower than the weight reported in several studies in other countries [20,33]. In a previous study, Sultana *et al*. [34] found the highest weight of 162.5 g/quail and an average weight of 145.8 g/bird experimentally in Bangladesh with different dietary nutrients supplementation. Our present findings coincide with several previous researchers who found an average body weight of 180-200 g at 5-6 weeks, and the females were heavier than males [23,35]. On the other hand, Ojo *et al*. and Seker *et al*. [22,36] reported that female birds were significantly heavier than those of male counterpart and mean quail birds’ body weights were 35.2 and 143.7 g, at 2^nd^ and 8^th^ weeks.

**Figure 3:**
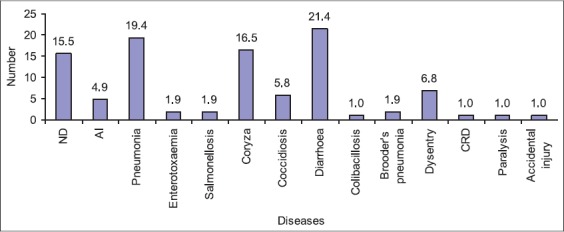
Prevalence of common diseases and disease conditions in quails (n=103 cases).

A larger proportion of quail farms (92.3%) has had the experience of disease prevalence, the proportion of healthy chick and death-in-shell was 92.1% and 13.5%, respectively (Table-4). Even though most of the farmers (73.0%) were well concerned about the general practice of biosecurity measures, only 52.0% farmers took veterinary care or advice whenever needed. Although a larger proportion of quail farms (92.3%) have had the experience of disease prevalence, in most cases (75.0%), the prevalence was less frequent. Diarrhea (21.4%) was identified as most prevalent diseases in these farms followed by pneumonia (19.4%), infectious coryza (16.5%), Newcastle disease (15.5%), dysentery (5.8%), and avian influenza (4.9%) (Figure-3). The proportions of healthy chick and death-in-shell were 92.1% and 13.5%, respectively. Improvement in fertility could be achieved by improving environmental conditions and early embryonic mortality (death in shell) can significantly affected by breed, variety, size, shape of eggs, and prevalence of diseases in parent stock farms [35,36]. Fertility in Japanese quails can be affected by different factors such as mating ratio, parental age, rate of laying, climatic and management conditions, and younger birds represented higher significant percentages of fertility compared to those from older layer [13,37-39]. Although Japanese quails are comparatively more resistant to infectious diseases than chickens, some infectious diseases such as salmonellosis, coccidiosis, infectious coryza, enteric diarrhea, and pneumonia have been reported in different earlier studies and in our current study.

**Table-4:** Disease management practices in selected quail farms.

Criteria	Groups	n (%)
Disease problem in the farm	Yes	48 (92.3)
	No	4 (7.6)
Disease frequency	Less frequent	39 (75.0)
	Rare	13 (25.0)
Percent of shell death	13.5%	
Percent of healthy chick	92.1%	
Percent of unhealthy chick	6.6% (1.5-20)	
Percent day-old-chick died	2.8% (1-8)	
Vet/consultant’s advice	Regularly	1 (1.9)
	At intervals	18 (34.7)
	Whenever needed	27 (52.0)
	Never	6 (11.5)
Bio-security practices	Strictly followed	6 (11.5)
	Generally followed	38 (73.0)
	Never followed	8 (15.3)

**Figure 4:**
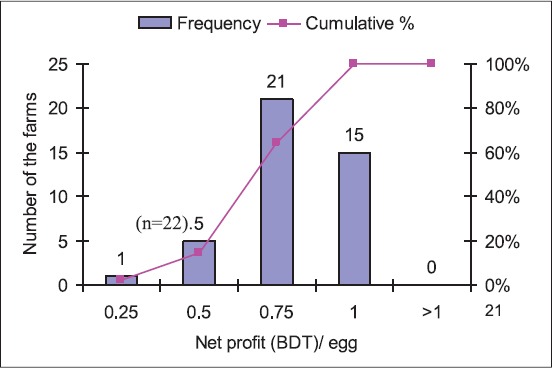
Frequency distribution of net profit (BDT) per egg in layer farms (n=42 farms).

**Figure 5:**
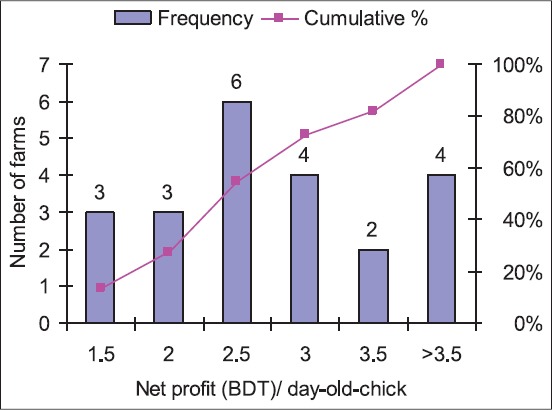
Frequency distribution of net profit (BDT)/day-old-chick in the quail hatcheries (n=22).

**Figure 6:**
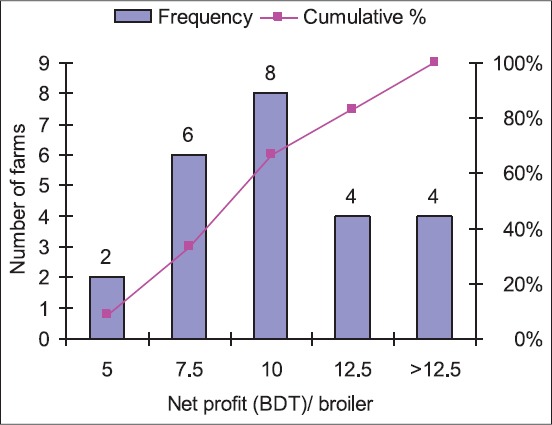
Frequency distribution of net profit (BDT)/broiler in broiler quail farms (n=24).

This study revealed ~50.0% layer farms had net profit of BDT 0.75/egg. However, the net profit was BDT 1.00/egg in 35.0% farms and BDT 0.25-0.50/egg only in 15.0% farms. The minimum, maximum and average net profits were BDT 0.10, 1.00 and 0.69, respectively (Figure-4). In the case of parent stock farming, 58.0% hatcheries had net profit of BDT 2.00-3.00/selling of a day-old-chick. The net profit of per day-old chick was BDT 1.50 in 14.0% hatcheries, BDT 3.50-3.50 in 28.0% hatcheries. The minimum, maximum and average net profits were BDT 1.50, 4.00 and 2.75, respectively (Figure-5) and were higher in parent stock farming than the layer farming. Broiler quail farming was found most profitable in Bangladesh, and net profit per broiler was ranged from BDT 4.00 to 13.00 with an average of BDT 9.02. The net profit/broiler was BDT 5.00 in 8.0% farms, BDT 7.50 in 25.0% farms, BDT 10.00 in 34.0% farms, and BDT ≥12.5 in 33.0% farms (Figure-6).

In spite of having enormous potentials of quail farming in Bangladesh has got many constraints. This study revealed that higher price (mean BDT 39.9/kg) of poultry feed (94.2%) and outbreak of recent endemic diseases (92.3%) were the major constraints for sustainable quail farming in Bangladesh. However, other constraints were lack of proper knowledge (80.8%), difficulties in collection of parent stock (78.8%), lack of proper market access by the farmers (71.2%), lack of farmers training (67.3%), inadequate bio-security practices (88.2%), and limited access to veterinary care (52.0%) (Table-5). The high cost of feeds is one of the major problems of commercial poultry production including Japanese quails and its cost usually ranged between 65% and 75% of the total production cost in Bangladesh [40].

**Table-5:** Major constraints found in selected farms that limit the raising of quails.

Causes	Number of farms (%)
Lack of proper knowledge	42 (80.8)
Higher feed price	49 (94.2)
Collection of parent stock elsewhere other than farmers owns farm	41 (78.8)
Lack of proper market access	37 (71.2)
Lack of farmers training	35 (67.3)
Outbreak of endemic diseases	48 (92.3)
Inadequate bio-security practices	46 (88.3)
Limited access to veterinary care	27 (52.0)

## Conclusion

Recognizing the enormous potentiality of Japanese quail as an alternative to chickens in providing gainful employment, supplementary income and as a valuable source of meat and egg, quail farming should be encouraged and promoted in Bangladesh. However, this study has explored the very poor situation and major constraints of Japanese quail farming throughout the country which brought about many challenges to the researchers, academicians and practicing veterinarians to adopt all the strategies to make quail farming economically and commercially viable in near future in Bangladesh.

## Authors’ Contributions

ANMAR was the principal investigator of the project and designed the project and experiments, visited most of the quail farms, collected significant portions of the data and prepare the manuscript. MNH collected some of the data and helped in manuscript preparation and editing. AKT helped in the analysis of the data and ZCD helped in manuscript editing.

## References

[1] Rahman M (2000). Bangladesh poultry men need help. Poult. Int.

[2] Das S. C, Chowdhury S. D, Khatun M. A, Nishibori M, Isobe N, Yoshimura Y (2008). Poultry production profile and expected future projection in Bangladesh. World's Poult. Sci. J.

[3] Nakamura R. M (1990). A Livestock and Poultry Disease Control Program for Bangladesh. Checchi/USAID/BRAC, Dhaka.

[4] Haque M. E, Howlider M. A. R, Huque Q. M. E (1999). Growth performance and meat yield characteristics of native naked-neck and their crosses with exotic chicken. J. Appl. Anim. Res.

[5] Onyewuchi U. U, Offor I. R, Okoli C. F (2013). Profitability of quail bird and egg production in IMO state. Nig. J. Agric. Food Environ.

[6] Saidu S, Afanasyev G, Popova L, Komarchev A, Ibrahim U (2014). Dynamic of Reproductive Qualities of Japanese Quails. International Conference on Earth, Environmentand Life Sciences, (EELS-2014) December 23-24, Dubai (UAE).

[7] Minvielle F (2004). The future of Japanese quail for research and production. W. Poult. Sci. J.

[8] Rahman M. S, Rasul K. M. G, Islam M. N (2010). Comparison of the productive and reproductive performance of different color mutants of Japanese quails (*Coturnix japonica*).

[9] Ojedapo L. O, Amao S. R (2014). Sexual dimorphism on carcass characteristics of Japanese quail (*Coturnix coturnix japonica*) reared in derived Savanna zone of Nigeria. Int. J Sci., Environ. Technol.

[10] Karapetyan R (2003). Biological and efficiency quality of quails. B. Bird.

[11] Faitarone A. B. G, Pavan A. C, Mori C, Batista L. S, Oliveira R. P, Garcia E. A, Pizzolante C. C, Mendes A. A, Sherer M. R (2005). Economic traits and performance of Italian quails reared at different cage stocking densities. Braz. J. Poult. Sci.

[12] Aygun A, Sert D (2013). Effects of prestorage application of propolis and storage time on eggshell microbial activity, hatchability, and chick performance in Japanese quail (*Coturnix coturnix japonica*) eggs. Poult. Sci.

[13] Jatoi A. S, Sahota A. W, Akram M, Javed K, Hussain J, Mehmood S, Jaspal M. H (2013). Response of different body weights on blood serum chemistry values in four close-bred flocks of adult Japanese quails (*Coturnix coturnix japonica*). Pak. J. Zool.

[14] Kalsum U, Soetanto H Achmanu, Sjofjan O (2012). Influence of a Probiotic containing Lactobacillus fermentum on the laying performance and egg quality of Japanese quails. Int. J. Poult. Sci.

[15] Tunsaringkarn T, Tungjaroenchai W, Siriwong W (2013). Nutrient benefits of quail (*Coturnix coturnix japonica*) eggs. Int. J. Sci. Res. Pub.

[16] Narinc D, Aksoy T, Karaman E, Aygun A, Firat M. Z, Uslu M. K (2013). Japanese quail meat quality: Characteristics, heritabilities, and genetic correlations with some slaughter traits. Poult. Sci.

[17] Wahab M. A (2002). Quails could reduce protein deficiency in poor countries. W. Poult.

[18] Ophir A. G, Persaud K. N, Galef B. G (2005). Avoidance of relatively aggressive male Japanese quail (*Coturnix coturnix japonica*) by sexually experienced conspecific females. J. Comp. Psychol.

[19] Hoque M. A, Ali A, Bhuiyan A. K. F, Amin M. R (1996). Quantitative variation on of some economic traits in Japanese quail (*Coturnix coturnix japonica*). Bang. J. Anim. Sci.

[20] Egbeyale L. T, Fatoki H. O, Adeyemi O. A (2013). Effect of egg weight and oviposition time on hatchability and post hatch performance of Japanese quail (*Coturnix coturnix japonica*). Nig. J. Anim. Prod.

[21] Odunsi A. A, Rotimi A. A, Amao E. A (2007). Effect of different vegetable protein sources on growth and laying performance of Japanese quails (*Coturnix coturnix japonica*) in a derived savannah zone of Nigeria. W. Appl. Sci. J.

[22] Ojo V, Fayeye T. R, Ayorinde K. L, Olojede H (2014). Relationship between body weight and linear body measurements in Japanese quail (*Coturnix coturnix japonica*). J. Sci. Res.

[23] Singh N. P (2010). Quail Production and Management Technology *Personal communication* (Extension Folder No. 40/2010), ICAR Research Complex for Goa, Ela, India.

[24] Sultan A. J, Waheed A. S, Akram M, Javed K, Hussain J, Mehmood S, Hayat M. J (2013). Hatching traits as influenced by different body weight categories in four close-bred flocks of Japanese quails (*Coturnix coturnix japonica*). Pak. J. Zool.

[25] Ophir A. G, Galef B. G (2003). Female Japanese quail that 'eavesdrop'on fighting males prefer losers to winners. Anim. Behav.

[26] Mehdi B. G, Naser M. S, Alireza L, Ayub S. A (2010). Effects of setting eggs small end up on hatchability and embryo mortality in Japanese quail (*Coturnix coturnix japonica*). Glob. Vet.

[27] Yildirim I, Aygun A, Sert D (2015). Effects of preincubation application of low and high frequency ultrasound on eggshell microbial activity, hatchability, supply organ weights at hatch, and chick performance in Japanese quail (*Coturnix coturnix japonica*) hatching eggs. Poult Sci.

[28] Persaud K. N, Galef B. G (2003). Female Japanese quail aggregate to avoid sexual harassment by conspecific males: A possible cause of conspecific cueing. Anim. Behav.

[29] Gebreil O. S. R (2002). Effect of age, sex ratio, and male replacement on reproductive performance of quail. M.Sc. Thesis, Department of Animal Production.

[30] Narinc D, Aygun A, Sar T (2013). Effects of cage type and mating ratio on fertility in Japanese quails (*Coturnix coturnix japonica*) eggs. T. I. J. Agric. Sci. Dev.

[31] Brand H. V, Parmetier H. K, Kemp B (2004). Effects of housing system (out door vs cages) and age of laying hens on egg characteristics. Br. J. Poult. Sci.

[32] White D. J, Galef B. G (1999). Mate-choice copying and conspecific cueing in Japanese quail *Coturnix coturnix japonica*. Anim. Behav.

[33] Banerjee S (2010). Carcass studies of Japanese quails (*Coturnix coturnix japonica*) reared in hot and humid climate of Eastern India. W. Appl. Sci. J.

[34] Sultana F, Islam M. S, Howlider M. A. R (2007). Effect of dietary calcium sources and levels on egg production and egg shell quality of Japanese quail. Int. J. Poult. Sci.

[35] Magda I, Samaha A, Sharaf M. M, Hemeda S. A (2010). Phenotypic and genetic estimates of some productive and reproductive traits of Japanese quails. Egypt. Poult. Sci.

[36] Seker I, Ekmen F, Bayraktar M, Kul S (2004). The effects of parental age and mating ratio on egg weight, hatchability and chick weight in Japanese quail. J. Anim. Vet. Adv.

[37] Rizk R. E, Nadia A, El-Sayed E. H. A, Shahein H, Shalan M (2008). Relationship between egg shell, egg shell membranes and embryonic development through different egg production periods in two developed chicken strains. Egypt. J. Poult. Sci.

[38] Daikwo S. I, Dim N. I, Momoh M. O (2011). Hatching characteristics of Japanese quail eggs in a tropical environment. Int. J. Poult. Sci.

[39] Raji A. O, Mbap S. T, Kwari I. D (2015). Fertility and hatchability of Japanese quail eggs under semi arid conditions in Nigeria. Nig. J. Anim Prod.

[40] Haq A, Akhtar M (2004). Poultry Farming.

[41] Siddiqui S. A, Mondal M. A. S (1996). Economics of Japanese quail farming in Dhaka Metropolitan City. Bang J. Agric. Econ.

